# Cellular levels of Grb2 and cytoskeleton stability are correlated in a neurodegenerative scenario

**DOI:** 10.1242/dmm.027748

**Published:** 2017-05-01

**Authors:** Piyali Majumder, Kasturi Roy, Brijesh Kumar Singh, Nihar Ranjan Jana, Debashis Mukhopadhyay

**Affiliations:** 1 Biophysics and Structural Genomics Division, Saha Institute of Nuclear Physics, Block-AF, Sector-1, Bidhannagar, Kolkata, West Bengal 700064, India; 2 Cellular and Molecular Neuroscience Laboratory, National Brain Research Centre, Manesar, Gurgaon 122 050, India

**Keywords:** Alzheimer's disease, APP/PS1 mouse model, Growth factor receptor bound protein 2, GRB2, Cytoskeleton, β-amyloid, β-amyloid protein precursor intracellular domain, AICD

## Abstract

Alzheimer's disease (AD) manifests as neuronal loss. On the premise of Grb2 overexpression in AD mouse brain and brain tissues of AD patients, our study primarily focuses on the stability of cytoskeletal proteins in the context of degenerative AD-like conditions. Two predominant molecular features of AD, extracellular accumulation of β-amyloid oligomers and intracellular elevation of amyloid precursor protein intracellular domain levels, have been used to closely inspect the series of signalling events. In their presence, multiple signalling pathways involving ROCK and PAK1 proteins lead to disassembly of the cytoskeleton, and Grb2 partially counterbalances the cytoskeletal loss. Increased Grb2-NOX4 interactions play a preventive role against cytoskeletal disassembly, in turn blocking the activity of nitrogen oxides and decreasing the expression of slingshot homolog 1 (SSH-1) protein, a potent inducer of cytoskeleton disassembly. This study unravels a unique role of Grb2 in protecting the cytoskeletal architecture in AD-like conditions and presents a potential new strategy for controlling neurodegeneration.

## Abstract (precis)

**Summary:** Grb2 has a unique role in protecting the cytoskeletal architecture in AD-like conditions, offering a potential new strategy for controlling neurodegeneration.

## INTRODUCTION

Alzheimer's disease (AD) is a progressive neurodegenerative disorder that destroys memory and thinking skills. The background molecular events proposed in the amyloid cascade hypothesis have been studied and restudied for years, but the exact relevance of the amyloid plaques to AD pathogenesis is still unclear and often doubted (De Strooper and Karran, 2016). Whilst β-amyloid (Aβ) plaques, some small and soluble oligomeric species of Aβ-like Aβ-derived diffusible ligands (ADDLs) (Haass and Selkoe, 2007; Klein et al., 2001; Townsend et al., 2006; Walsh et al., 2002) and neurofibrillary tangles (NFTs) remain the primary protagonists in AD, there has been growing interest in understanding the changes in the total cytoskeletal network through reorganization, sometimes grouped or referred to as cofilin pathologies (Bamburg and Bloom, 2009). Additionally, aberrant processing of amyloid precursor protein (APP) by β-secretase (BACE1) – generating cytotoxic APP intracellular domain (AICD) and Aβ_1-40_ or Aβ_1-42_ fragments (Selkoe, 1994) – has been shown to have consequential effects on neuronal and glial cells, ultimately leading to degeneration of the entire cytoskeleton network, including microfilaments, intermediate filaments and microtubules (Bamburg and Bloom, 2009; Liem and Messing, 2009). AICD-overexpressing transgenic mice have some of these typical AD pathological characteristics (Ghosal et al., 2009). These findings have led to the hypothesis that both Aβ peptide oligomers and AICD act synergistically, leading to cytoskeleton loss.

Of late, there has been growing interest to study non-amyloidogenic mechanisms like autophagy in neurodegenerative diseases. Recently, our group has shown that excess AICD under AD-like conditions is sequestered from the system by Grb2 through the autophagic pathway (Raychaudhuri and Mukhopadhyay, 2010; Roy et al., 2014). In general, Grb2, a cytoplasmic protein, plays a key role in Ras-mediated growth factor signalling, proliferation and the cell cycle. Several groups have established Grb2 as an important link between cellular signalling and the neuronal cytoskeleton (Lim and Halpain, 2000; Reynolds et al., 2008). Even during mitotic spindle formation, a requirement for phosphorylation of tau at specific residues, mediated by Grb2-ERK 1/2 signalling, has been shown (Nizzari et al., 2012). In control non-AD brains, Grb2 localizes all over the cell body and extends into dendrites, whereas in AD brains, the localization of Grb2 is restricted to the neuronal cell body (McShea et al., 1999; Raychaudhuri and Mukhopadhyay, 2007), where the intensity of its interaction with tyrosine-phosphorylated APP or with the C-terminal fragment (CTF) increases significantly (Russo et al., 2002; Venezia et al., 2004b) in neuronal cells and AD brains. This elevated interaction in turn elicits MAPK-mediated signalling and eventually apoptosis (Venezia et al., 2004a). The APP, CTF or AICD thus positively regulate apoptosis in the context of AD (Zhou et al., 2004). The signalling mediated by the Grb2 SH3 domain has also been studied and established in other stress situations. For example, Rom et al. (2011) have shown binding of the SH3 domain with HIV Tat, initiating an IGF1R-Raf-MAPK cascade. We have also reported previously that Grb2 is naturally overexpressed in different neurodegenerative scenarios, specifically Huntington's disease (HD), and that it shows a chaperone-like action there.

A major consequence of AD is the formation of NFTs comprising paired helical filaments (PHFs) made from hyperphosphorylated tau proteins. Tau is an intrinsic component of the neuronal cytoskeleton that is involved in microtubule assembly and stabilization in normal brains (Mandelkow and Mandelkow, 2012; Mokhtar et al., 2013). In addition to the formation of PHFs, there are reports of drastic degradation of other integral proteins of the cytoskeleton network. For example, the actin dynamics are reportedly altered in AD conditions through actin depolymerizing factor (ADF) and cofilin regulators. As mammalian neurons contain about 5- to 10-fold more cofilin proteins than ADF, the former is considered to be the more potent regulator of actin destabilization (Kuhn et al., 2000; Maloney et al., 2005; Minamide et al., 2000). In AD brains, upstream modulators of cofilin phosphorylation, like small RhoA, Rac1 GTPase, PAK1, and ROCK1 and ROCK2, are downregulated in the disease state, ultimately augmenting cofilin activity (Garvalov et al., 2007; Zhao et al., 2006). Vimentin, one of the intermediate filaments imparting structural stability and localization, is also disrupted in the AD neurons (Levin et al., 2009). The expression level of vimentin is significantly altered in AD (Chakrabarti et al., 2014). The anomalous pattern of events initiated in the AD brain suggests that once amyloid and tau pathologies start, neighbouring neuronal cells also be affected by synaptotoxicity of Aβ oligomers (Ferreira et al., 2015; García-Arencibia et al., 2010; Santa-Maria et al., 2012; Wang and Mandelkow, 2012). Complete understanding of cytoskeletal stability and its effects in AD is, therefore, important to elucidate the disease mechanism better.

In the present work, using a background of natural overexpression of Grb2 in AD mouse brain and in human AD brain lysates, we aimed to study the expression levels of cytoskeletal proteins in AD-like conditions along with the effects of Grb2 on them, and we discuss our results in the context of pathways that may be perturbed. We also examine how Grb2 might play a protective role in a cell's overall endeavour for survival.

## RESULTS

### Expression of Grb2 and several cytoskeletal proteins changes in human AD brain lysates and in brain tissue from an AD mouse model

**Fig. 1.**
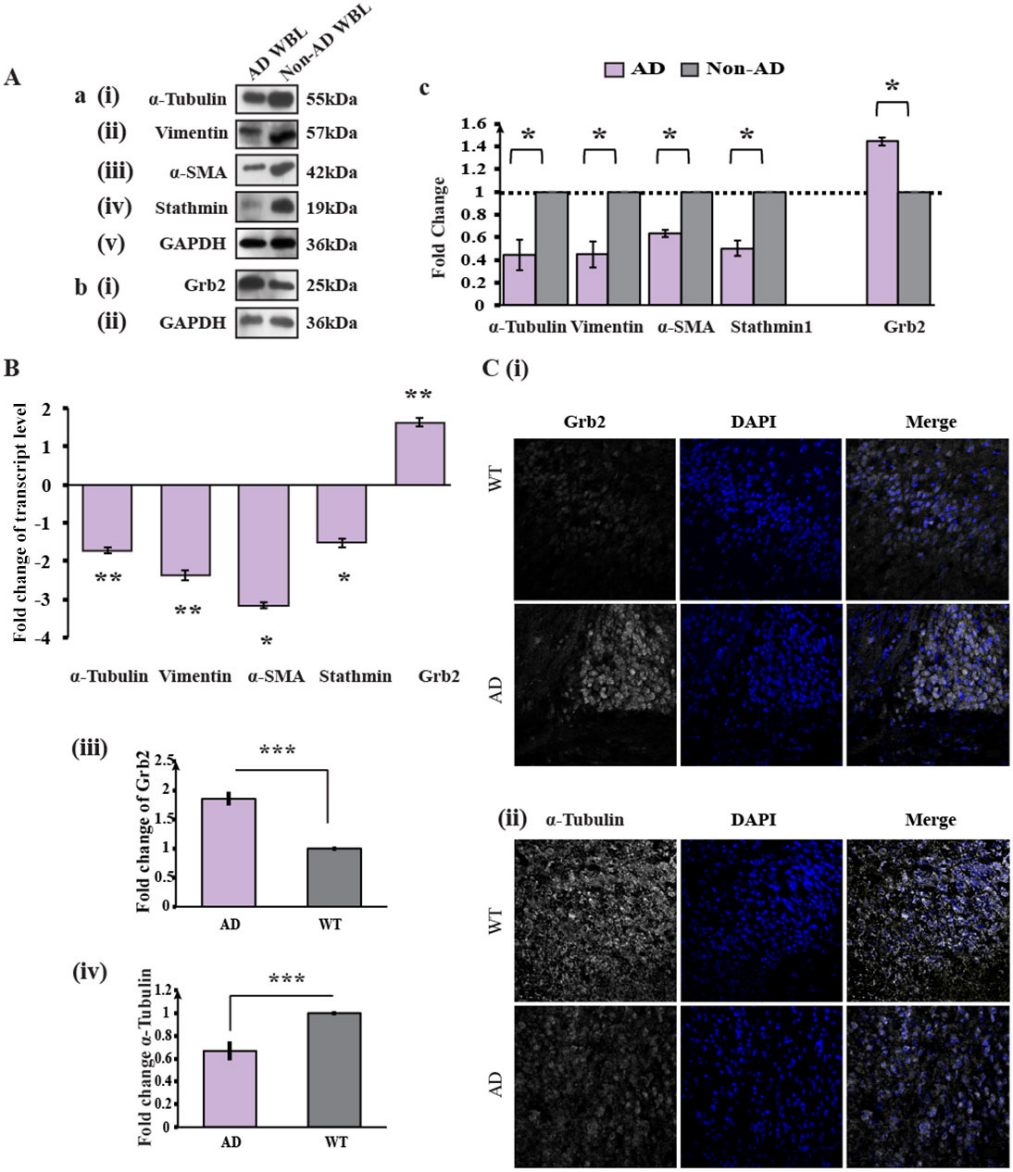
**Expression levels of cytoskeletal proteins decrease and those of Grb2 increase in AD whole brain lysates and the APP/PS1 mouse model.** (Aa) Representative western blots (*n*=2) of four cytoskeletal proteins – (i) α-tubulin, (ii) vimentin, (iii) α-SMA and (iv) stathmin 1 with (v) GAPDH used as an internal control in human AD whole brain lysates, compared to non-AD whole brain lysate. (Ab) Western blots (*n*=2) showing the (i) Grb2 and (ii) GAPDH levels in AD whole brain lysate. (Ac) Histogram representing the mean values of the optical density of the bands normalized against GAPDH, presented as the fold change relative to values in non-AD samples. (B) Transcript level changes in AD mouse brain tissue of genes encoding α-tubulin, vimentin, stathmin 1 and Grb2 by qRT-PCR, presented as the fold change relative to wild-type mouse brain tissue where it was normalized with internal control *Gapdh* gene. (C) Results of immunohistochemistry analysis for the expression of Grb2 and α-tubulin from paraffin-embedded sections of brain of AD mouse model and WT, where both Grb2 and α-tubulin staining was converted to greyscale, and the nucleus was stained with DAPI. Magnification, 60×. To gain a semi-quantitative analysis of the changes, pixel densities of the images were calculated using ImageJ software.

Recent studies have shown that in AD-like scenarios, the levels of several structural proteins are altered (Castaño et al., 2013; Chakrabarti and Mukhopadhyay, 2012; Chakrabarti et al., 2014; Morawski et al., 2013). We wanted to decipher the pathways that might be involved in the stability of the structural protein network and the possible role of Grb2 therein under disease conditions. The expression levels of Grb2 and four cytoskeletal proteins – α-tubulin, vimentin, α-smooth muscle actin (α-SMA) and stathmin1 – in human whole-brain post-mortem tissue lysates of AD patients and non-AD whole brain (control) tissue lysates were compared by western blotting (Fig. 1A). Grb2 was found to be overexpressed 1.44-fold in AD brains [Fig. 1Ab(i),Ac; *P=0.0064; *n*=2]. The relative levels of cytoskeletal proteins were found to be downregulated 2.23-fold [Fig. 1Aa(i),Ac; *P=0.0146; *n*=2] for α-tubulin, 2.21-fold [Fig. 1Aa(ii),Ac; *P=0.0061; *n*=2] for vimentin, 1.57-fold [Fig. 1Aa(iii),Ac;*P=0.007; *n*=2] for α-SMA and 1.99-fold [Fig. 1Aa(iv),Ac; *P=0.0177; *n*=2] for stathmin1. Similarly, the expression levels of all of the above four cytoskeleton proteins in the hippocampus lysate of an AD patient, in comparison to in a non-AD human hippocampus lysate (WT), showed similar trends of degradation (Fig. S1).

Transcript levels of *Grb2* and of mRNAs encoding four cytoskeletal proteins (α-tubulin, vimentin, α-SMA and stathmin1) were measured by performing quantitative real-time PCR (qRT-PCR) (Fig. 1B) for an AD mouse model. Under AD conditions, Grb2 expression showed significant (**P=0.0002) upregulation compared to WT, and cytoskeletal proteins showed significant (**P=0.0003 α-tubulin; **P=0.001 vimentin; *P=0.013 α-SMA and *P=0.0192 stathmin1) downregulation. To validate the changes visually, immunohistochemistry (IHC) for one representative protein, α-tubulin, was performed on APP/presenilin 1 (PS1) mice brain sections [B6C3-Tg(/APPswe,PSEN1dE9/) 85Dbo/J mice]. Marked visual differences were noted at the expression levels of Grb2 [Fig. 1C(i) and C(iii)] and of the cytoskeletal proteins α-tubulin [Fig. 1C(ii) and C(iv)] in AD mouse brain sections. Semi-quantitative comparisons of pixel densities showed a significant increase for Grb2 (***P<0.0001, 30 cells from three independent samples) and a significant decrease for α-tubulin (***P<0.0001, 30 cells from three independent samples).

### Expression of Grb2 and cytoskeletal proteins also changes in human neuroblastoma cells under AD-like conditions

**Fig. 2.**
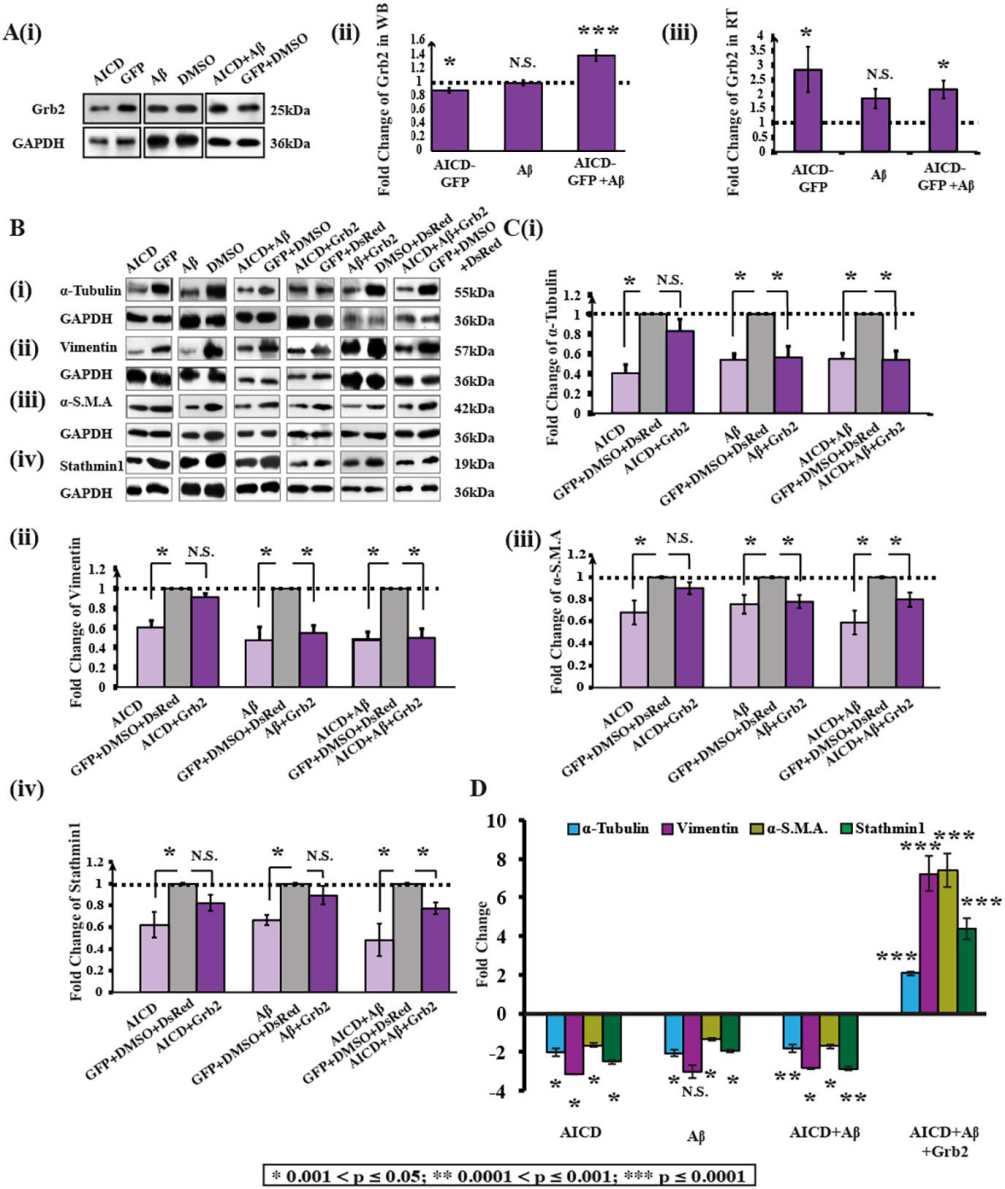
**Grb2 is upregulated and cytoskeletal proteins are downregulated in an AD-mimicking neuroblastoma cell model.** (A)(i) Western blot (*n*=3) showing alterations of Grb2 and GAPDH levels in AICD-transfected, Aβ-treated and both AICD-transfected and Aβ-treated cells compared with respective negative controls (empty vector or solvent used for reagent). (ii) Graphical representation of the normalized variation of Grb2 expression compared to control cells (set as 1) (transfected with GFP, treated with DMSO and both transfected with GFP and treated with DMSO, respectively). (iii) Normalized fold changes of mRNA levels of Grb2 for qRT-PCR experiments (*n*=3), presented as a fold change relative to values in respective negative controls (empty vector or solvent used for reagents), with GAPDH taken as an internal control. (B) Western blots depict alterations in the expression of (i) α-tubulin, (ii) vimentin, (iii) α-smooth muscle actin (α-SMA) and (iv) stathmin 1 with GAPDH. The samples are derived from the same experiments and the blots were processed in parallel. (C) Histograms showing changes in the four cytoskeletal proteins respectively in (i-iv). Fold changes relative to controls are presented. (D) Shows transcript level changes for the four cytoskeletal proteins under AICD, Aβ and AICD+Aβ conditions with or without Grb2.

To mimic an AD-like environment, we transiently transfected human neuroblastoma cells (SHSY-5Y) with an AICD-GFP construct and treated them externally with Aβ peptide (Aβ_1-42_) for 48 h. Despite significant upregulation in Grb2 transcript levels detected using qRT-PCR [Fig. 2A(iii)], in the presence of both the AD-inducing factors (AICD and Aβ), the expression of Grb2 protein was, however, higher in comparison to that upon treatment with AICD or Aβ alone [Fig. 2A(i),A(ii)] as western blotting demonstrated that AICD and Aβ were unable to increase Grb2 protein levels individually. When AICD was transiently transfected into cells, the Grb2 protein level was decreased 1.13-fold (*n*=3; *P=0.0017) and the transcript level was increased 2.85-fold (*n*=5; *P=0.04<0.05). Whereas, for Aβ-like conditions, Grb2 protein level (1.01-fold change; *n*=3; *P*=0.75) and transcript level (1.56-fold change; *n*=5; *P*=0.12) changes were not significant. In the case of simultaneous treatment with AICD and Aβ, protein and transcript levels increased significantly 1.38-fold (*n*=3; ***P=0.0001) and 2.16-fold (*n*=5; *P=0.0021), respectively.

In this disease model, both protein and transcript levels of α-tubulin [Fig. 2B(i),C(i),D], vimentin [Fig. 2B(ii),C(ii),D], α-SMA [Fig. 2B(iii),C(iii),D] and stathmin1 [Fig. 2B(iv),C(iv),D] were found to be decreased significantly. Individually, all the conditions – the cellular presence of AICD or extracellular presence of Aβ, or both – showed significant deterioration in the assembly of each cytoskeletal protein. These results are summarized in Table 1. These cell model data were found to be in accordance with both AD brain lysate data and mouse AD model data.

**Table 1.**
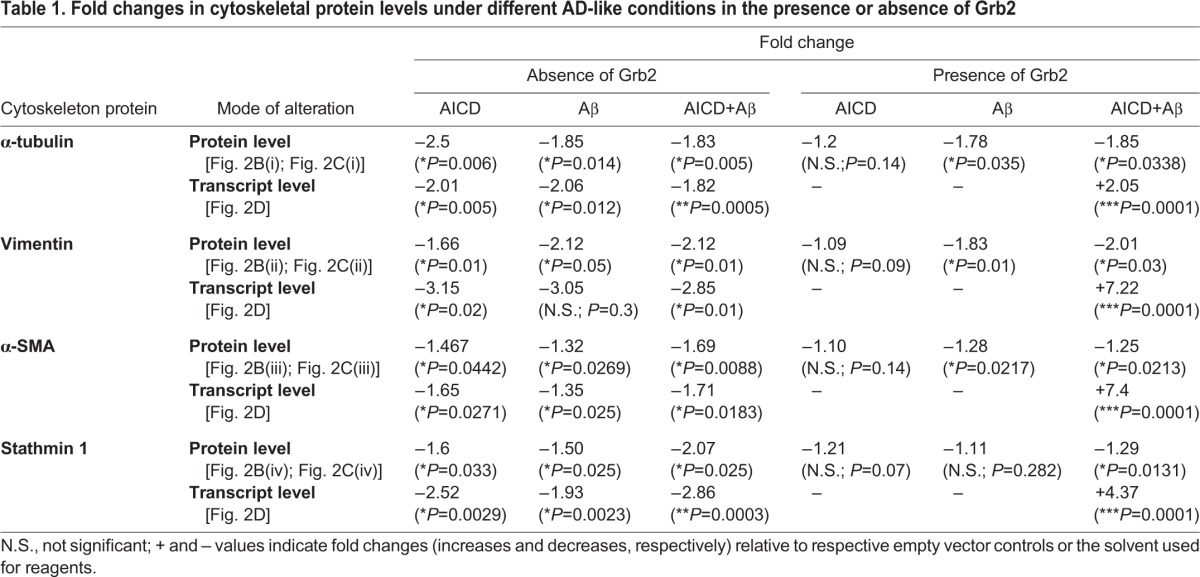
**Fold changes in cytoskeletal protein levels under different AD-like conditions in the presence or absence of Grb2**

### Grb2 partially reverses the effects of AD-like pathological stress on cytoskeletal proteins

Given that Grb2 is naturally overexpressed in whole brain lysates of AD patients and also that it sequesters AICD in vesicles (McShea et al., 1999; Raychaudhuri and Mukhopadhyay, 2010; Roy et al., 2014), it was reasonable to hypothesize that Grb2 might reverse the cytoskeletal protein degradation in AD-like conditions. Restoration in both protein and transcript levels of the cytoskeleton proteins were investigated upon Grb2 overexpression [Fig. 2B,C(i),C(ii),C(iii),C(iv),D]. The effects of Grb2 on four cytoskeletal proteins, α-tubulin, vimentin, α-SMA and stathmin1 under AICD+Grb2, Aβ+Grb2 and AICD+Aβ+Grb2 conditions, in contrast to transfection with their respective empty vectors, were significant (see Table 1).

From Table 1, the percentage recovery of cytoskeletal proteins from disassembly upon Grb2 administration could be estimated. In AICD-overexpressing conditions, Grb2 upregulated the expression of α-tubulin, vimentin, α-SMA and stathmin1 by 71.6%, 77.75%, 69.17% and 53.56%, respectively. Whereas, for Aβ-treated conditions, Grb2 overexpression downregulated those four cytoskeletal protein levels by 4.5%, 14.1%, 10.58% and 68.25%, respectively. Likewise, in AICD-transfected and Aβ-treated conditions, Grb2 intervention increased the expression of α-tubulin, vimentin, α-SMA and stathmin1 by 1.09%, 5.09%, 51.1% and 55.7%, respectively. At the transcript level, presence of Grb2 upregulated the transcript levels of α-tubulin, vimentin, α-SMA and Stathmin1 significantly by 212.6%, 353.94%, 710.13% and 195.78%, respectively.

To further substantiate our findings, we also checked the consequences under Grb2 knockdown conditions in which we transfected SHSY-5Y cells with a U6-Grb2 construct and showed that knocking down Grb2 significantly increased the disassembly of most of the cytoskeleton proteins (i.e. α-tubulin, α-SMA and stathmin1), except for that of vimentin (Fig. S2).

### Cytoskeletal proteins disassemble under AD-like stress

**Fig. 3.**
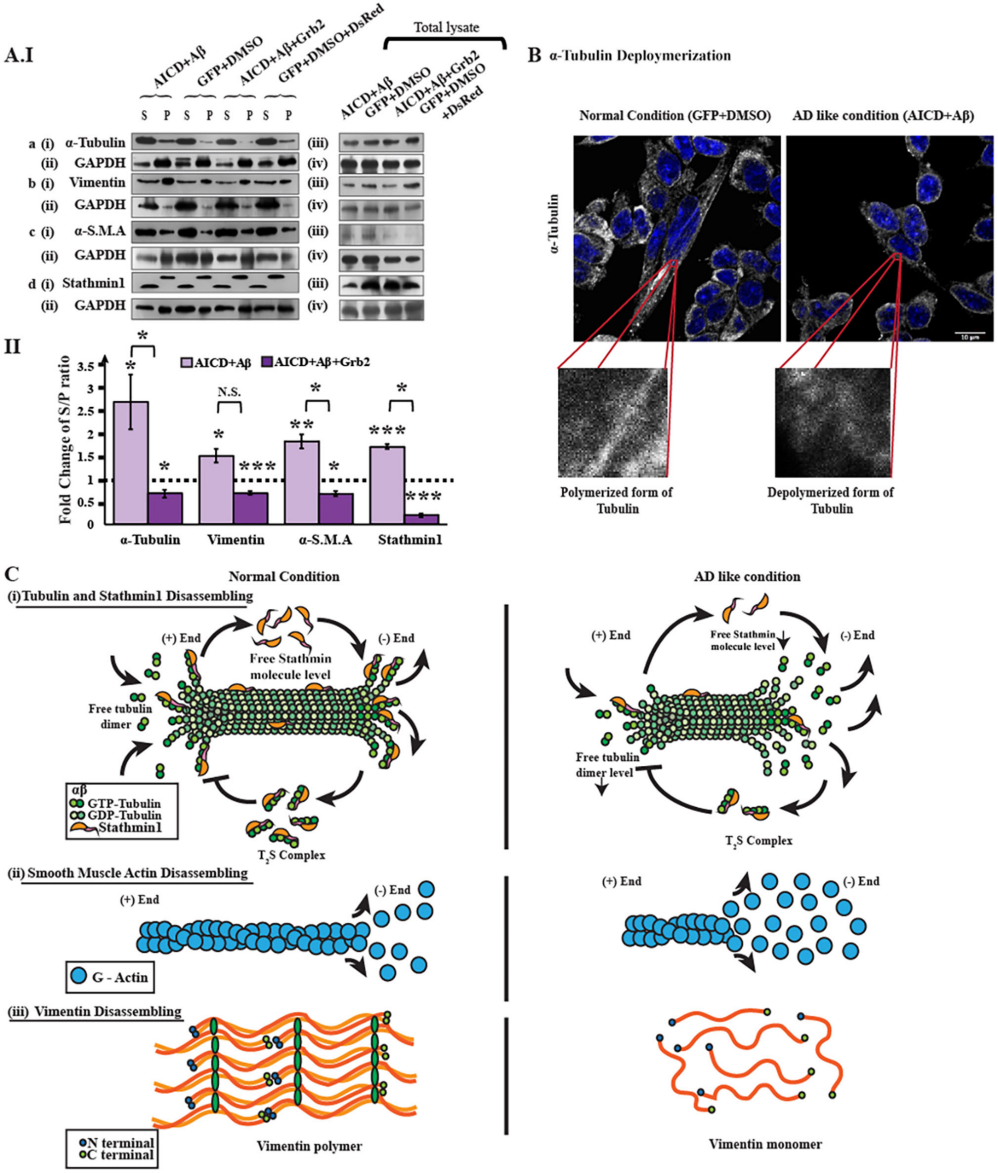
**Disassembly assay of cytoskeletal proteins.** The microtubule network comprises α- and β-tubulin monomers, and many microtubule-associated proteins (MAPs). Stathmin1 is one such MAP. Similarly, actin polymers are also composed of globular actin (G-Actin) monomers. As α-smooth muscle actin (α-SMA) is nothing but a variant of actin, it is disassembled in a manner similar to that of general actin polymers. Vimentin is a stack of aggregated vimentin peptide monomers, which have a distinct pattern. (AIa) Western blots represent (i) α-tubulin in the supernatant and pellet fractions for disease-inducing (AICD and Aβ) and reversal (AICD, Aβ and Grb2) conditions, along with (ii) an internal control GAPDH. (iii),(iv) Alterations of α-tubulin in total cell lysate. Western blots for other three cytoskeletal proteins – vimentin, α-SMA and stathmin 1 – are also depicted in AIb(i-iv), AIc(i-iv), AId(i-iv), respectively. (AII) Bar diagram demonstrating the alterations in disassembling factors (S/P ratio) for α-tubulin, vimentin, α-SMA and stathmin 1. Values are presented as the fold change relative to negative controls (empty vector or the solvent used for reagents). (B) Immunocytochemistry result depicting disassembly of α-tubulin polymer tracks in AICD-transfected and Aβ-treated cells. α-tubulin is shown in grey, and the nucleus is marked with DAPI staining. Zoomed images of polymer tracks for both conditions are given in the inset box. (C) Cartoon representations of molecular events of disassembly of (i) tubulin and stathmin 1, (ii) α-smooth muscle actin (α-SMA) and (iii) vimentin, respectively.

We investigated the mechanism of cytoskeletal protein degradation after 48 h of (A) both AICD transfection and the addition of Aβ (AD-inducing condition) or (B) both AICD transfection and Aβ treatment along with Grb2 overexpression (AD reversal condition). In each case, we measured the disassembling factors for the cytoskeletal proteins, expressed as an S/P ratio where ‘S’ stands for the amount of protein in the supernatant fraction and ‘P’ denotes the amount of protein in the pellet fraction (Fig. 3A). It was found that the S/P ratio increased significantly (S/P>1) for all the four proteins in condition A, where fold increases of 2.7, 1.5, 1.8 and 1.7 were calculated for α-tubulin, vimentin, α-SMA and stathmin 1, respectively. However, under condition B, the situation was reversed (S/P<1), and fold changes for S/P ratios were −1.43, −1.46, −1.47 and −4.9 for α-tubulin, vimentin, α-SMA and stathmin 1, respectively. Although stathmin 1, being a microtubule-associated protein (MAP), was not expected to show any self-assembly; however, upon disassembly of the microtubule network, stathmin 1 became detached from the network, and we could distinguish free unattached stathmin 1 in the supernatant from the microtubule-attached fraction in the pellet. Stathmin 1 showed a different pattern compared to those of other cytoskeletal proteins [Fig. 3C(i)]. The pellet fraction of Stathmin1 (S) showed a higher molecular mass compared to that in the supernatant fraction because of tubulin monomers (α and β) were attached to stathmin 1 (forming a complex, referred to as T_2_S). Depolymerization of α-tubulin was shown in SHSY-5Y cells by immunocytochemistry (Fig. 3B), where α-tubulin was fragmented in AICD- and Aβ-transfected cells compared to control GFP-transfected and DMSO-treated cells. The modalities of disassembly for α-tubulin, α-SMA and vimentin are depicted in the cartoon [Fig. 3C(i)-C(iii)].

### Multiple signalling pathways lead to cytoskeleton degradation

**Fig. 4.**
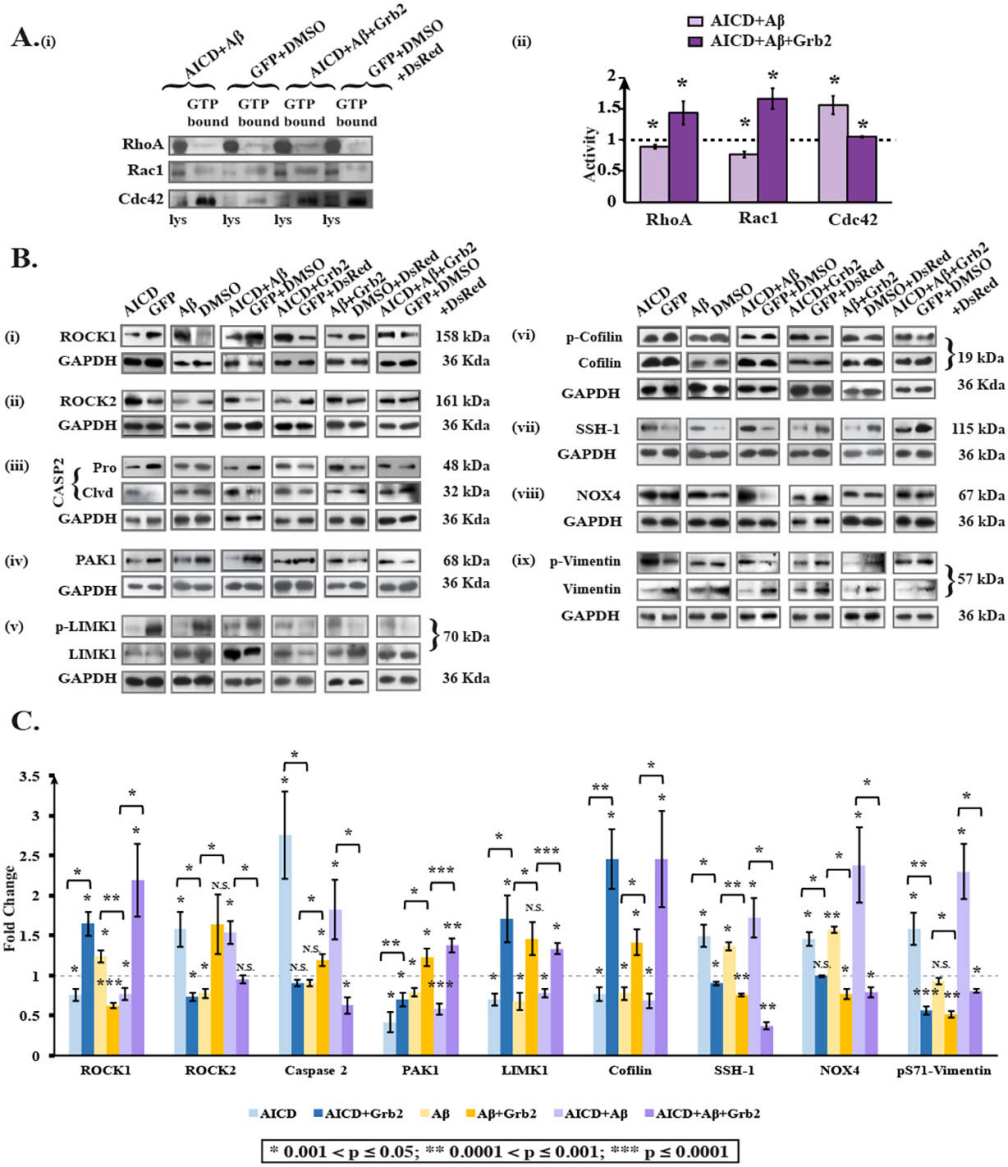
**Signalling molecules participate in AD-like conditions.** (Ai) Western blot for activity changes of small GTPases – i.e. RhoA, Rac1 and CDC42 under AD-inducing (AICD+Aβ) and reversal conditions (AICD+Aβ+Grb2). (ii) Bar diagram represents the activity alterations for the small GTPases. (B) Protein levels and activation of signalling molecules – (i) ROCK1, (ii) ROCK2, (iii) Caspase 2, (iv) PAK1, (v) LIMK1, (vi) cofilin, (vii) SSH-1 and (viii) NOX4 – by western blotting. (ix) Changes in levels of phosphorylated vimentin at serine 71 are shown; these serve an important role in signalling. (C) Graphical representation of these alterations of signalling molecules and phosphorylated vimentin (pS71-vimentin). Values are shown as fold changes relative to negative controls (empty vector or the solvent used for reagents). p-, phosphorylation of the indicated protein.

While investigating the pathways that could be responsible for degradation of cytoskeletal proteins, we found an involvement of three small GTPases – RhoA, Rac1 and Cdc42 [Fig. 4A(i),A(ii)]. Under AICD-transfected and Aβ-treated disease-inducing conditions, activities of RhoA and Rac1 were decreased significantly 1.12- (*n*=3) and 1.3-fold (*n*=3), respectively, whereas the activity of Cdc42 was increased 1.55-fold (*n*=3). The effect was reversed in the presence of Grb2, and RhoA, Rac1 and Cdc42 activity levels were increased 1.43- (*n*=3), 1.65- (*n*=3) and 1.04-fold (*n*=3), respectively, instead. These alterations in the activities of small GTPases were sufficient to perturb the downstream signalling events and the cytoskeletal proteins degraded primarily through ROCK- and PAK1-mediated pathways (Koleske, 2013; Salminen et al., 2008). To understand these effects, we categorized our subsequent experiments into (A) a disease-inducing condition, where cells were transiently transfected with AICD and treated with Aβ and (B) a reversal condition, in which AICD and Grb2 were transiently co-transfected and cells were treated with Aβ, and finally, effects on downstream pathways were monitored.

Under the AD-inducing condition A, the downstream effectors of RhoA – ROCK1 and ROCK2 – showed opposing outcomes in changes in their expression; ROCK1 decreased 1.3-fold (*n*=4) and ROCK2 increased 1.54-fold (*n*=4). However, on reversal (condition B), the 2.19-fold increase of ROCK1 (*n*=4) was much higher than the insignificant 1.05-fold increase of ROCK2 (*n*=4) [Fig. 4B(i),B(ii),C]. We also checked for ROCK2 activity as well as its expression level under both conditions A and B, and we found that ROCK2 activity was increased 1.3-fold (*n*=3) under condition A, and in condition B, activity was decreased 1.52-fold (*n*=3) [Fig. S3(i)-S3(iii)]. Caspase 2, another upstream effector of ROCK2 besides RhoA (Ho et al., 2008; Vakifahmetoglu-Norberg et al., 2013), was upregulated under condition A 1.825-fold (*n*=3) but downregulated 1.597-fold (*n*=3) under condition B [Fig. 4B(iii),C]. Expression of PAK1, the downstream effector for both Rac1 and Cdc42 (Koth et al., 2014; Zhao et al., 2006) and upstream regulator of stathmin1, was reduced in scenario A 1.73-fold (*n*=3) but increased in scenario B 1.37-fold (*n*=3) [Fig. 4B(iv),C]. Interestingly, both ROCK isoforms and PAK1, an activator of LIMK1 by phosphorylation (Cuberos et al., 2015; Mendoza-Naranjo et al., 2012), expectedly reduced LIMK1 activity under condition A 1.28 fold (*n*=4) and activated it significantly under condition B 1.335-fold (*n*=4) [Fig. 4B(v),C]. Cofilin proteins, downstream effectors of LIMK1 whose dephosphorylation enables actin severing and depolymerizing activities (Huang et al., 2006), exhibited a 1.46-fold (*n*=4) reduction in activity under condition A, and their activity was highly increased under condition B 2.45-fold (*n*=4) [Fig. 4B(vi),C]. Expression of the cofilin dephosphorylating protein SSH-1 (Woo et al., 2015) was elevated significantly in condition A 1.72-fold (*n*=3) and was reduced greatly 2.70-fold (*n*=3) under condition B [Fig. 4B(vii),C], which probably intensified the observed activities of cofilin proteins. Additionally, the distal upstream effector of SSH-1, NOX4 (Brown and Griendling, 2009; Kim et al., 2009; Xi et al., 2013), was overexpressed 2.38-fold (*n*=3) under condition A, whereas under condition B, its expression was significantly reduced (1.26-fold) [Fig. 4B(viii),C].

Additionally, destructive phosphorylation of serine 71 in vimentin (Goto et al., 1998) was increased 2.3-fold (*n*=4) in condition A, whereas in reversal conditions (B), it was reduced 1.24-fold (*n*=4) [Fig. 4B(ix),C]. Under control conditions when cells were (1) transfected with AICD alone, (2) co-transfected with AICD and Grb2, (3) treated with Aβ alone or (4) transfected with Grb2 and externally treated with Aβ, serine 71 phosphorylation levels on vimentin increased 1.58-fold (*n*=3), and decreased 1.78- (*n*=3), 1.07- (*n*=3) and 1.96-fold (*n*=3), respectively. Considering that α-tubulin was downregulated in AD-like conditions [Fig. 2B(i),C], we checked the activity of Gsk3β, a kinase that hyperphosphorylates tau, leading to destabilization of the microtubule network, and found that its activity was increased significantly 1.17-fold under condition A but decreased significantly 1.46-fold under condition B [Fig. S4(i),(iii)], whereas the activity of its upstream effector AKT was decreased significantly 2.44-fold under condition A [Fig. S4(i),(ii)]. All the subtle changes in expression and activities are summarized in Table 2.

**Table 2.**
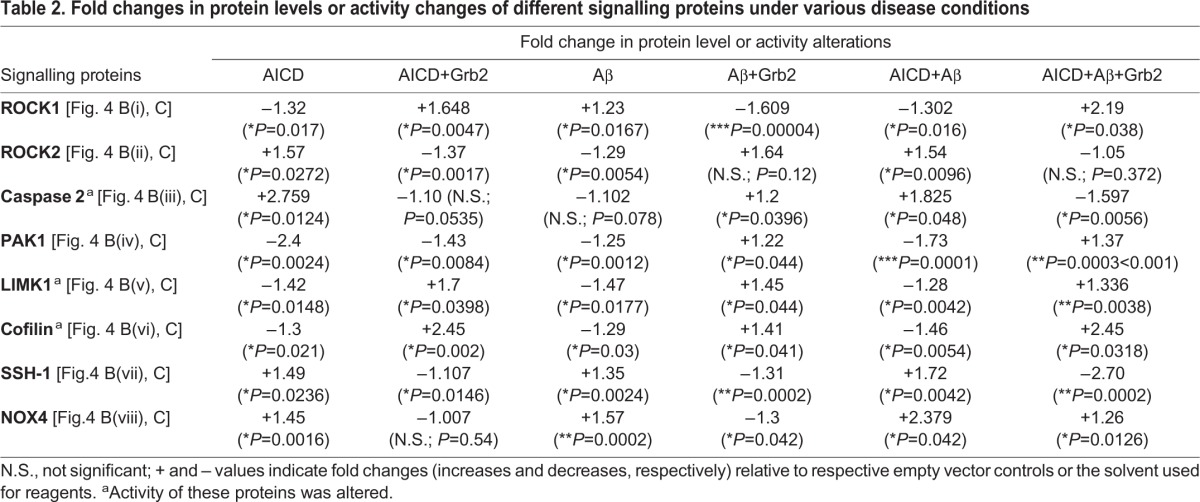
**Fold changes in protein levels or activity changes of different signalling proteins under various disease conditions**

### Increased Grb2-NOX4 interactions play a preventive role in cytoskeletal disassembly

**Fig. 5.**
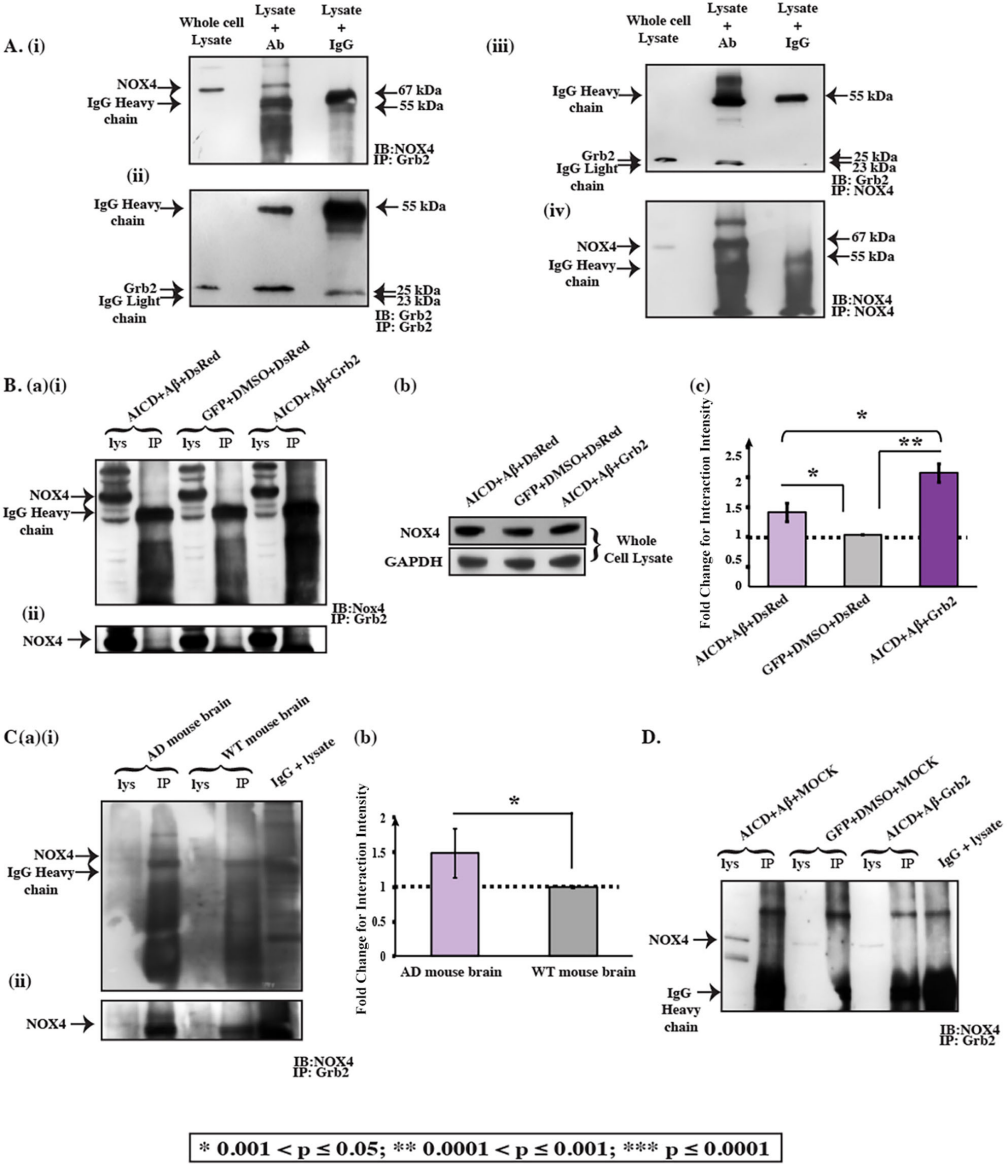
**The Grb2 and NOX4 interaction prevents cytoskeletal degradation by decreasing the availability of NOX4.** (A) Co-immunoprecipitation (Co-IP) blot where immunoprecipitation (IP) was performed for Grb2, and then precipitates were immunoblotted (IB) with an anti-NOX4 antibody (i) and anti-Grb2 antibody (ii). Reverse Co-IP blot where immunoprecipitation was performed for NOX4, and then precipitates were probed with anti-Grb2 antibody (iii) and anti-NOX4 antibody (iv). (Ba) (i) The variation of the interaction of Grb2 and NOX4 under AD-inducing (AICD, Aβ and DsRed) and reversal (AICD, Aβ and Grb2) conditions compared to the controls (GFP, DMSO and DsRed). (ii) Higher exposure of the NOX4 bands from the same blot shown in (i). (Bb) Changes of NOX4 and GAPDH under AD-inducing and reversal conditions for whole cell lysate. (Bc) Graphical representation of the intensity of the interaction between NOX4 and Grb2, presented as fold changes relative to control (set as 1). (Ca) (i) Variation of the interaction between Grb2 and NOX4 in AD whole mouse brain lysate compared to WT control whole mouse brain lysate. (ii) A higher exposure of the NOX4 bands from the same blot shown in (i). (Cb) Graphical representation of the intensity of the interaction between NOX4 and Grb2, shown as the fold change relative to WT brain (set as 1). A greater interaction was seen under the AD condition compared to under the WT condition. (D) The interaction of NOX4 and Grb2 under Grb2 knock down. The Co-IP experiment shows that the NOX4 and Grb2 interaction decreases compared to under the AD-like situation, in which both AICD and Aβ were present in abundance. Lys, whole lysate.

In reversing the signalling pathways involved in the degradation of cytoskeletal proteins, Grb2 was found to play a pivotal role. Abnormal NOX activation has been reported in AD (Hernandes and Britto, 2012). NOX4 was found to interact with Grb2 to activate Src (Xi et al., 2013). Interestingly, NOX4 was found to be overexpressed endogenously 2.379-fold in an AD-like cell model but reduced in the presence of Grb2 [Fig. 4B(viii),C]. The interaction of Grb2 with NOX4 was enhanced 1.42-fold under AD conditions (Fig. 5B). Transfection with Grb2 further enhanced the NOX4-Grb2 interaction, to 2.18-fold relative to control. The effect of NOX4 recruitment and its expression inhibition by Grb2 was somewhat cumulative, reducing its relative availability for other cellular functions. This might also corroborate the decrease in cofilin phosphatase activity of SSH-1 to 2.70-fold [Fig. 4B(vii),C]. Furthermore, we checked the intensity of the interaction of NOX4 and Grb2 in AD mouse brain lysate, which increased significantly (1.4875-fold) in the AD context compared to in the wild type [Fig. 5C(a),(b)]. The interaction decreased in the Grb2 knock down conditions, where SHSY-5Y cells were transfected with a U6-Grb2 siRNA construct (Fig. 5D). Similarly, upon NOX4 knockdown, the cofilin phosphatase SSH-1 expression decreased by 28.46% and its downstream α-tubulin increased by 61.5%, reversing the disassembly (Fig. S6).

Additionally, although overexpressing Grb2 could significantly reverse the effect of many AD pathologies, to our surprise in the working AD-like model, Grb2 not only failed to reduce the activity level of reactive oxygen species (ROS) but, rather, elevated it 1.27-fold [Fig. S5(i),(ii)].

## DISCUSSION

Emerging evidence reveals the inadequacies of the ‘amyloid cascade hypothesis’ for AD (Pimplikar, 2009). Although for decades this hypothesis depicts Aβ plaques as the major constituents of AD (Hardy and Selkoe, 2002), the linearity of the cascade remains controversial. In a recent review, De Strooper and Karran (2016) emphasize that progressive neurodegeneration in AD probably takes place through the accumulation of pathological alterations at different phases of the cell. For stabilizing the homeostatic pressure of this progressive degeneration, the cell itself upregulates some factors that supposedly revert these pathological alterations to some extent. Based on our previous observations, we hypothesized Grb2 as one such stabilizing factor for AD-like pathological stress.

Our group first demonstrated in HD, another progressive neurodegenerative disease, that elevated expression of Grb2 in HD animal and cell models reduces aggregation of mutant Huntingtin (Htt) protein, the hallmark of HD (Baksi et al., 2013). Several reports on Grb2 strongly suggest its role in survival in AD through sequestration of AICD via autophagy (Raychaudhuri and Mukhopadhyay, 2010; Roy et al., 2014). The present study for the first time demonstrates that Grb2 is naturally overexpressed in AD whole human brain lysate and also in the APP/PS1 AD mouse model [Fig. 1A,B,C(i),(iii)], probably as a causality to reinforce cellular survival in AD. To mimic AD at the basic level, we developed a system in which major constituents of AD, like AICD and Aβ, were present in abundance. In this system, simultaneous transfection of AICD followed by Aβ treatment in human neuroblastoma cells showed significant upregulation of Grb2 at both protein and transcript levels. The Aβ oligomers added externally to the medium are known to take up conformations like those of ADDLs (Dahlgren et al., 2002). Nevertheless, between individual components of AD-inducing factors, only AICD could elicit a change of Grb2 levels significantly (Fig. 2A). From this observation, we could extend our explanation that being a trans-activator, AICD is able to cause upregulation of Grb2, whereas the external addition of Aβ failed to achieve the same effect.

Given that the death of a neuron is an ultimate fate in AD, the collapse of cytoskeletal protein architecture could be correlated with disease advancement, although not in a linear way. Some direct pieces of evidence also corroborate the link of cytoskeletal proteins with AD pathology – (i) the fibrillar structure of NFTs, hallmarks of AD, comprising hyperphosphorylated tau, which ultimately destabilizes the tubulin network (Terry, 1998); (ii) PS1, a major component of the γ-secretase complex, can interact with δ-catenin, eventually influencing the restructuring of the actin cytoskeleton (Koutras et al., 2011; Levesque et al., 1999); and (iii), in addition, AD brains also contain rod-shaped structures called Hirano bodies, comprising actin and an actin-associated cofilin, the destruction of which explicitly affects cytoskeletal network (Yao et al., 2010). We have shown in AD whole brain lysates, as well as in an AD mouse model and in AICD-transfected and Aβ-treated neuroblastoma cells, that several cytoskeletal and associated proteins have deficits in expression at both the transcript and protein levels [Fig. 1A-C(ii),(iv); Fig. 2B-D]. In these contexts, however, transcript levels of these cytoskeletal components increased several fold with overexpression of Grb2 in conjunction with the abundance of both AICD and Aβ (Fig. 2D), the latter being the predominant event in AD brains and the APP/PS1 AD mouse model. This result could be validated at the protein level, at least in the situation where both Grb2 and AICD were in abundance. These point towards an overall perturbation of the cytoskeletal network in our cell-based model of AD (Fig. 3A,B). The cartoon in Fig. 3C clearly depicts this alteration in α-tubulin, stathmin1 and α-SMA, which disassemble from a polymer, and in vimentin, which disintegrates under the disease condition, as these factors showed S/P values >1. They reconstitute the network again upon Grb2 overexpression in the disease condition, when S/P values become <1.

**Fig. 6.**
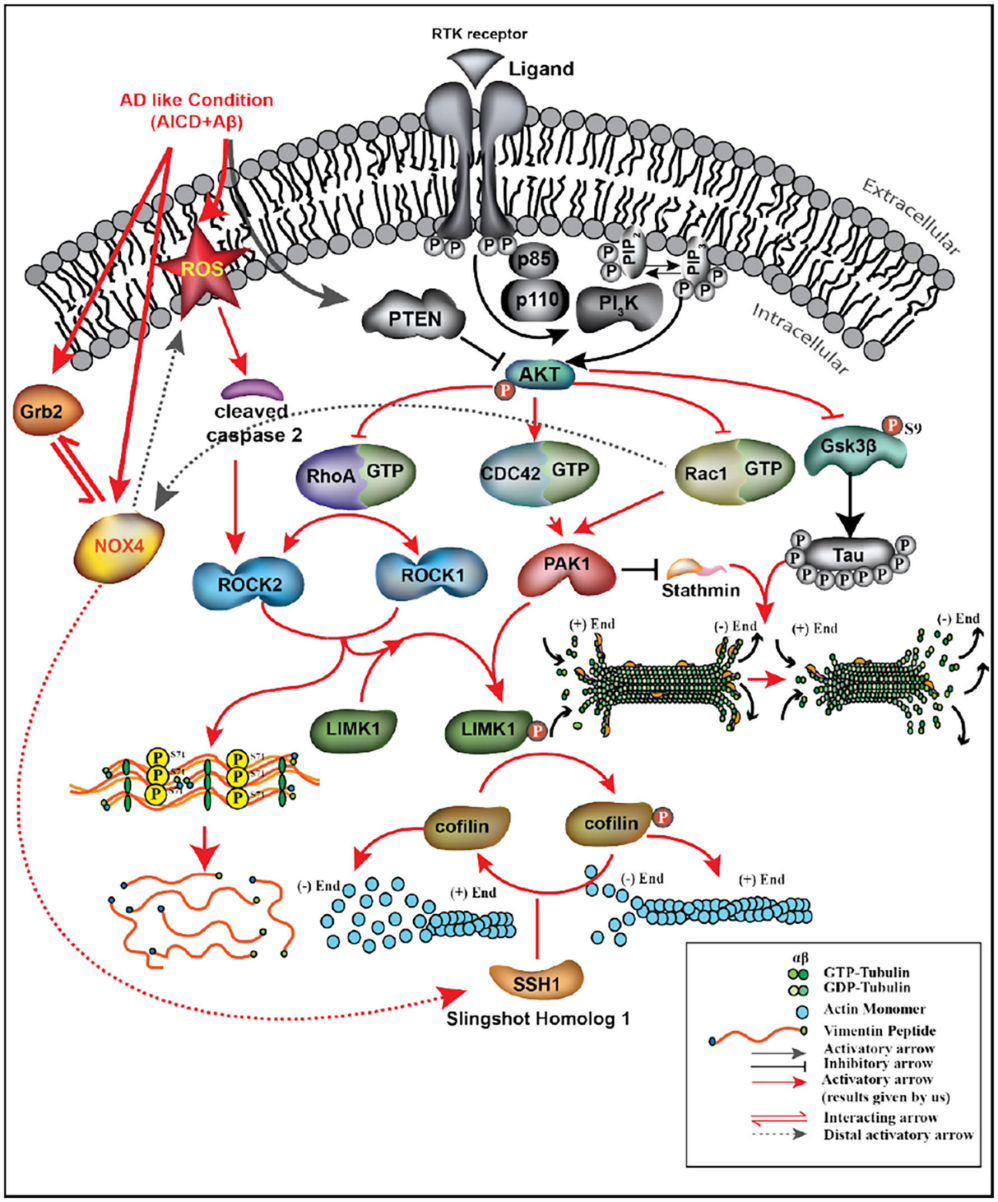
**Representation of the molecular events occurring in cells under AD conditions, where AICD and Aβ are upregulated and Grb2 is overexpressed Grb2.** Results obtained by us are represented in colour.

It is prudent therefore to try to understand the underpinning cellular mechanisms that help in the cytoskeletal restructuring, with Grb2 playing the pivotal role (Fig. 6). Three small GTPases – RhoA, Rac1 and Cdc42 – are known to regulate cell shape changes through rearrangement of cytoskeletal proteins (Bolognin et al., 2014; Jeanteur, 2012; Spiering and Hodgson, 2011), acting as molecular switches. We found a significant decrease in RhoA and Rac1 and an increase in Cdc42 activity, all of which were reversed in the presence of Grb2 [Fig. 4A(i),(ii)]. A similar increase in RhoA has been reported in SHSY-5Y cells by Petratos et al. (2008), supporting our assumption that the simultaneous abundance of Aβ, AICD and Grb2 in and around these cells could be a true reflection of an AD-like condition. Elevation of Cdc42 activity is justified as it is known to be agonistically regulated with autophagy by phosphatidylethanolamine-conjugated LC3 proteins (LC3II) (Chung et al., 2012), and our group has previously demonstrated autophagy induction in a similar context (Roy et al., 2014). Considering that Rac1 activity decreases significantly in our model, the expression of PAK1, the downstream effector of both Cdc42 and Rac1, relies on the fine balance of the activities of both. We found a significant decrease in PAK1 expression that was reversed upon Grb2 administration [Fig. 4B(iv),C]. In a similar way, with a drop in RhoA activity, ROCK1 expression decreased but ROCK2 expression increased. Upon overexpression of Grb2, ROCK1 [Fig. 4B(i),C] level increased significantly, whereas that of ROCK2 [Fig. 4B(ii),C] was only marginal. Commensurate to that, ROCK2 activity also followed the same trend (Fig. S3). Considering that ROCK2 is a substrate for caspase 2, whose expression also increased significantly in our AD model, this apparent discrepancy can be adequately addressed (Herskowitz et al., 2013) [Fig. 4B(iii),C]. Continuing with these subtle shifts in balance along the signalling cascade, the extent of phosphorylation and hence the activity of LIMK1, the downstream effector of both PAK1 and ROCK isoforms, show a strong dependence on the combined effect of ROCK1, ROCK2 and PAK1. We show that notwithstanding the significant dip in LIMK1 activity as a combined effect of its antecedents, the reversal in activity in the presence of Grb2 was not pronounced [Fig. 4B(v),C]. In spite of that, cofilin, the most crucial regulator of actin dynamics (Minamide et al., 2000), was significantly activated in our system, and upon intervention with Grb2, was inactivated through phosphorylation [Fig. 4B(vi),C]. The expression of SSH-1 and its distal upstream effector NOX4 increased significantly in the disease model. Interestingly, the first kinase phosphorylates cofilin and the second dephosphorylates it (Huang et al., 2006; Woo et al., 2015) and expression of both is significantly decreased upon Grb2 overexpression [Fig. 4B(vii),C]. As expected, when Grb2 and NOX4 were knocked down in our model, reverse effects were recorded on cytoskeleton assembly. For most of the cytoskeletal proteins upon Grb2 knock down, the extent of perturbations were more than the combined effects of AICD and Aβ. Grb2 overexpression could reverse this effect.

In the wake of these small imbalances at different levels along the signalling cascades, culminating in a large-scale perturbation in the cytoskeleton network, the role of Grb2 emerged as that of reversing cell fate to a large extent. While estimating the contributions of other concurrent effects due to Grb2 overexpression that affected cytoskeletal integrity, we noted that NOX4 [Fig. 4B(viii),C], a ROS-activating protein, had already been reported to interact with Grb2 under normal conditions (Montenegro et al., 2015; Xi et al., 2013). We show in our study that this Grb2-NOX4 interaction was increased several fold under disease conditions and, with an increase in Grb2 expression, this interaction was further promoted (Fig. 5A,B). Recruitment of NOX4 by Grb2 would expectedly disrupt its availability for activation of its downstream SSH-1, which would, in turn, lead to phosphorylation of cofilin and finally degradation of the actin network. As NOX4 is responsible for ROS generation, we also checked whether Grb2 overexpression could regulate ROS, and found that it could not [Fig. S5(i),(ii)]. On the contrary, ROS activity was increased in the presence of excess Grb2, AICD and Aβ. This might be a hint towards the fact that even though the cells geared up the protective mechanism, the defence was lost with the progression of the disease.

In AD, the microtubule network degrades mostly upon hyperphosphorylation of tau protein (Bamburg and Bloom, 2009; Mokhtar et al., 2013) as a result of loss of the microtubule stabilizing activity of tau. Tau is hyperphosphorylated by many protein kinases, and one such kinase is Gsk3β, whose activity was found to be upregulated in our disease model and downregulated in the presence of Grb2 [Fig. S4(i),(iii)]. The upstream inhibitor of Gsk3β is AKT1, whose activity was downregulated expectedly in the presence of Grb2 [Fig. S4(i),(ii)].

Intermediate filament vimentin was also degraded in our AD cell model. ROCK isoforms can phosphorylate vimentin on serine 71, and this unique phosphorylation is known to reorganize vimentin and inhibit filament formation (Goto et al., 1998). In this study, significant elevation of phosphorylation on serine 71 of vimentin in AICD-transfected and Aβ-treated disease conditions was noted, which decreased significantly upon Grb2 overexpression [Fig. 4B(ix),C]. Similarly, stathmin1 expression, which was significantly decreased in our model, reverted upon Grb2 overexpression. Incidentally, stathmin1 is known to reduce in AD, and its protein level negatively correlates with the amount of tangle but not with plaque quantity (Cheon et al., 2001) [Fig. 2B(iv),C(iv),D]. Additionally, it is important to note that our experiments were performed after 48 h of introduction of AD-mimicking parameters. The cells’ intention to survive under AD-like pathological stress is clear. The crux of the study boils down to the ubiquitous role played by Grb2 in restoring cytoskeletal degradation by interfering with the signalling at different levels. It is premature to apprehend anything about the retroactive failure of the process, however. Understanding the influence of other fate-determining factors that could play a critical role in tilting the homeostatic balance toward degradation of the cytoskeleton and its associated proteins remain to be deciphered.

## MATERIALS AND METHODS

### Ethics statement

All animal experiments were conducted following the institutional guidelines for the use and care of animals and approved by the Institutional Animal and Ethics Committee of the National Brain Research Centre (NBRC/IAEC/2012/71).

### Plasmids, whole brain lysate and chemicals

AICD was cloned into a pGFP-C1 vector (Clontech) and Grb2 into a DsRed-C1vector (BD Biosciences). Here, we describe AICD-GFP construct as ‘AICD’ and similarly, Grb2-DsRed construct as ‘Grb2’. An siRNA construct against Grb2, U6-Grb2 was generated as explained in Raychaudhuri and Mukhopadhyay (2010), and siRNA against NOX4 was purchased from Ambion – Life Technologies, cat. no. 4392420, siRNA ID #s224161. Aβ peptide was purchased from Sigma-Aldrich (A980). AD (NB820-59363) and non-AD (NB820-59177) post-mortem whole brain lysates were purchased from Novus Biologicals. For statistical reasons, products from different patients were used: for product # NB820-59363, brain lysate of 75-year-old Caucasian male, known pathology is cause of death by AD (lot # B105129) and a 72-year-old Caucasian male, known pathology of cause of death also AD (lot # B909049), whereas for product # NB820-59177, non-AD brain lysate of 60-year-old Caucasian female (Lot # B811092), cause of death is unknown but patient had emphysema and a 98-year-old Caucasian male (Lot # C101138), cause of death was prostate cancer. We also purchased hippocampus brain lysate of both AD (cat. # ab30181, lot # GR190367-5; age, 93 years; cause of death, lymphoma; disease state, AD brain sample) and control (cat # ab30180 with, lot # GR258239-1; age, 82 years; cause of death, aortic stenosis; disease state, normal brain sample). All the patient details and disease stage etc. have been compiled in Tables S1 and S2.

### APP/PS1 mice

APP/PS1 or B6C3-Tg(/APPswe,PSEN1dE9/)85Dbo/J mice were obtained from the Jackson Laboratory and maintained in the institute's animal house facility. These transgenic mouse lines for AD express human APPswe mutations (K670N and M671L) and exon-9-deleted human presenilin 1(PSEN1dE9) under the control of the mouse prion gene promoter. Animals were provided water and food *ad libitum*. The genotyping was performed using PCR as described previously (Ghate et al., 2014).

AD mice, along with controls at their ages of 8 and 12 months, were anaesthetized with xylazine (10 mg/kg body weight) and ketamine (100 mg/kg body weight) and perfused transcardially with PBS followed by 4% paraformaldehyde (w/v) in PBS. Brains were collected and further placed in 4% paraformaldehyde for 24 h and then treated with 10, 20 and 30% sucrose (in PBS) followed by sectioning in a freezing microtome (20 μm thickness). Sections (both control and AD) were placed on the same slides.

### Cell culture and transfection

Human neuroblastoma, SHSY-5Y, cells were obtained from National Cell Science Centre, Pune, India, and were cultured in DMEM-F12 (Gibco) supplemented with 10% fetal bovine serum (Gibco) at 37°C under a 5% CO_2_ atmosphere under humidified conditions. Transfection of cells with different constructs, like pGFP C1, DsRed, AICD-GFP or Grb2-DsRed was performed using Lipofectamine 2000 Transfection Reagent (Invitrogen). In the case of co-transfection, constructs were added in equal proportions. After 48 h, transiently transfected cells were checked for transfection efficiency by monitoring GFP or DsRed expression under the fluorescence microscope and were then used for experiments.

### Immunohistochemistry

Immunohistochemistry was performed on formalin-fixed paraffin-embedded tissue sections (20 μm), as previously described (Coleman and Stanley, 1994), by using antibodies against the following proteins at manufacturer-recommended dilutions: Grb2 and α-tubulin (Abcam, # ab32037 and ab4074, respectively. ImageJ software was used for the calculation of the intensity correlation quotient.

### Aβ_1-42_ protein fragment treatment

Aβ_1-42_ protein fragment (Sigma, A980) is neurotropic and also neurotoxic. It was added to the medium at a concentration of 0.5 µM 3 h after transfection, and samples were collected 48 h after addition (Dahlgren et al., 2002).

### RNA isolation, c-DNA preparations and real-time PCR

RNA was isolated from SHSY-5Y cells using the TRIzol reagent (Invitrogen, USA) extraction method following the manufacturer's protocol. RNA samples were quantified using a Nanodrop 2000 spectrophotometer (Thermo Scientific). RNA equivalent to 2 to 1 μg was taken to synthesize the first-strand cDNA using oligo dT primers (Fermentas) and reverse transcriptase (Invitrogen). Real-time RT-PCR reaction was performed using Sybr green 2× Universal PCR Master Mix (Applied Biosystems) with an ABI Prism 7500 sequence detection system. Primer sequences and PCR conditions are given in Table S3. The absolute quantification given by the software was in terms of CT values. The relative quantification of target genes was obtained by normalizing with an internal control gene (*GAPDH* gene).

### Protein from mammalian cells

PBS-washed pellets from cell lines were lysed on ice in lysis buffer (1 M Tris-HCl, pH 7.5, 1 M NaCl, 0.5 M EDTA, 1 M NaF, 1 M Na_3_VO_4_, 10% SDS, 20 mM PMSF, 10% Triton X-100, 50% glycerol) for 30 min in the presence of complete protease inhibitor (Roche Diagnostics) and centrifuged at 13,000 ***g*** for 15 min. Protein concentration was determined by using a Bradford protein estimation assay.

### Protein from paraffin-embedded tissue

Protein was isolated from paraffinized tissue sections of AD and WT mouse brains, as described previously (Guo et al., 2012) by using extraction buffer. Co-immunoprecipitation experiments were then performed where Grb2 pull down samples were probed with anti-NOX4 antibody. Antibodies are described below.

### Western blot

The cell lysate was separated on SDS gels according to molecular mass, then it was transferred to PVDF membrane (Millipore Corporation), which was blocked with 5% skimmed milk in TBST (50 mM Tris-HCl, 150 mM NaCl, pH 7.5, containing 0.05% Tween 20). After that, the membrane was probed with primary antibody, followed by incubation with horseradish peroxidase (HRP)-conjugated secondary antibody. The immunoreactive bands in the membrane were then developed with ECL kit (Super Signal West Pico Substrate; Pierce or Abcam). Quantification of western blots was performed using Quantity One software (Bio-Rad). At least three separate experiments were analyzed, and band intensities were normalized to a loading control. *P*-values were determined using an unpaired *t*-test. The lack of effects of the vehicles in transfection was confirmed (Fig. S7).

### Antibodies

Antibodies against Grb2 (ab32037) (1:5000 dilution), α-tubulin (ab4074) (1:5000 dilution), vimentin (ab8069) (1:5000 dilution), α-SMA (ab32575) (1:2500 dilution), stathmin1 (ab52630) (1:10,000 dilution), ROCK1 (ab45171) (1:5000 dilution), ROCK2 (ab125025) (1:5000 dilution), caspase 2 (ab32021) (1:1000 dilution), vimentin phosphorylated on serine 71 (ab115189) (1:1000 dilution), NOX4 (ab133303) (1:1000 dilution), slingshot homolog-1 (SSH-1) (ab76943) (1:1000 dilution), phosphorylated cofilin (S3) (ab12866) (1:1000 dilution), phosphorylated AKT1 (at serine 473) (ab81283) (1:1000 dilution), AKT1 (ab124341) (1:1000 dilution), phosphorylated Gsk3β (S9) (ab75814) (1:1000 dilution), Gsk3β (ab32391) (1:1000 dilution) and GAPDH (ab9484) (1:5000 dilution) were from Abcam. However, cofilin (CST-3318), phosphorylated LIMK (at threonine 508) (CST-3841s) (1:1000 dilution), total LIMK1 (CST-3842) (1:1000 dilution) and an antibody recognising PAK1, PAK2 and PAK3 (CST-2604) (1:1000 dilution) were from Cell Signaling Technology.

### Separation of cytoskeletal proteins into assembled and disassembled fractions

Whole cell lysates were taken from 90 mm culture dishes with 1 ml lysis buffer (50 mM HEPES, pH 7.4, 150 mM NaCl, 1% NP-40, 0.5% sodium deoxycholate, 0.1% SDS) and incubated at 41°C for 20 min. Soluble and insoluble fractions of cell lysates were separated by ultracentrifugation at 75,000 rpm for 1 h (rotor TLA 120.1, Beckman Coulter). A portion of the lysate (1/15th of the total) was saved before centrifugation, which was used as a whole cell lysate. The soluble fraction (S) and insoluble fractions (P) were collected, and the pellet was then resuspended in Laemmli buffer (one-third of the volume of initial lysis buffer used). Equal volumes of supernatant and pellet fractions were then analyzed by western blotting (Srivastava and Chakrabarti, 2014).

### Immunocytochemistry

SHSY-5Y cells that had been transfected with AICD-GFP or Grb2-DsRed, or with both AICD-GFP and Grb2-DsRed, were fixed with 4% formaldehyde and immunostained. Cells were permeabilized using 10% FBS in PBS with 0.1% saponin (Sigma Aldrich) for 60 min, followed by overnight staining in primary antibody at 4°C and 60 min incubation in secondary antibody at room temperature. The samples were then imaged using a confocal microscope (Nikon AIR+Tie with N-SIM and FCS microscope system). Cell boundaries were marked using ImageJ software, where images were converted to 8 bit, thresholded and mean intensity values were recorded using the ‘measure’ option.

### RhoA, Rac1 and Cdc42 activation assay

For the RhoA, Rac1 and Cdc42 activation assay, the Cytoskeleton (cat. no. BK030) RhoA/Rac1/Cdc42 Activation Assay Combo BiochemKit was used as per the manufacturer's protocol. In short, freshly prepared cell lysate, around 1 mg of protein, was added to 30 μl of rhotekin-RBD (Rho-binding domain) beads for the RhoA activation assay or 10 μl of PAK-PBD (p21-binding domain) beads for the Rac1 and Cdc42 activation assays. Mixtures were incubated on a rocker for 1 h at 4°C. Beads were then pelleted down by centrifugation at 5000 ***g*** at 4°C for 1 min. Very carefully, supernatant was removed, and the beads were washed with 500μl of wash buffer. Again, after centrifugation at 5000 ***g*** at 4°C for 3 min, the supernatant was removed and beads were boiled in 20 μl of Laemmli buffer (125 mM Tris-HCl, pH 6.8, 20% glycerol, 4% SDS, 0.005% Bromophenol Blue, 5% β-mercaptoethanol). Samples were then analyzed by western blotting.

### ROCK activity assay

ROCK2 activity was measured by using the ROCK Activity Immunoblot Kit (Cell Biolabs Inc.; cat. no. STA-415) following the manufacturer's protocol. According to the protocol, cell lysate from a 90-mm culture dish was used as the ROCK sample. To initiate the reaction, 25 µl of lysate was added to 50 µl of a mixture of 1× kinase buffer, ATP and MYPT1 protein (ROCK2 substrate) and incubated at 30°C with gentle agitation. The reaction was then stopped by addition of 25 µl of 4× reducing SDS-PAGE sample buffer. After boiling, 20 µl of sample was used for western blotting. The blot was probed with an anti-phosphorylated-MYPT1^Thr696^ anti-rabbit antibody, which was provided with the kit.

### Fluorescence-activated cell sorting and ROS activity

Cells were transfected with AICD-GFP and/or Grb2-DsRed, and then treated with Aβ peptide; a suitable control  of empty vector and treatment with DMSO was also performed. After 48 h, SHSY-5Y cells were harvested and stained with 5-(and-6)-chloromethyl-2′,7′ dichlorodihydrofluoresceindiacetate acetyl ester (CM-H_2_DCFDA) according to the manufacturer's protocol. The cells were then analyzed for ROS activity by fluorescence-activated cell sorting scan flow cytometry (BD FACS Calibur platform, California, USA).

### Statistical analysis

The mean s.d. was calculated using Microsoft Excel. For statistical analysis, an unpaired ‘*t*’ test was performed to compare the means of two experimental groups using the online software GraphPad Quick Cals, available at http://www.graphpad.com/quickcals/ttest.cfm. The error bars represent s.e.m. [(standard deviation/√*n*); *n*=sample size]. Statistical significance is shown with asterisks: *P≤0.05; **P≤0.001; ***P≤0.0001; N.S., not significant. To arrive at the statistically significant sample size for each experiment, we performed power analysis using a previously described model (Cohen, 1988), as incorporated in the G*power 3.1 (Faul et al., 2009) software using the following formula:
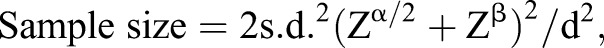


where, s.d., standard deviation; Z^α/2^ and Z^β^ are type 1 and 2 errors, respectively; d=effect size=difference between mean values. In the worst possible scenarios, we kept the type 1 error to 7% and type 2 error to 80% so that the power was always above 85%.
